# Evaluation of the immune response of peripheral blood mononuclear cells cultured on Ti6Al4V-ELI polished or etched surfaces

**DOI:** 10.3389/fbioe.2024.1458091

**Published:** 2024-10-08

**Authors:** Hugo Abreu, Mari Lallukka, Davide Raineri, Massimiliano Leigheb, Mario Ronga, Giuseppe Cappellano, Silvia Spriano, Annalisa Chiocchetti

**Affiliations:** ^1^ Department of Health Sciences, Interdisciplinary Research Center of Autoimmune Diseases-IRCAD, Università del Piemonte Orientale, Novara, Italy; ^2^ Center for Translational Research on Autoimmune and Allergic Diseases-CAAD, Università del Piemonte Orientale, Novara, Italy; ^3^ Applied Science and Technology Department, Politecnico di Torino, Torino, Italy; ^4^ Orthopaedics and Traumatology Unit, “Maggiore della Carità” Hospital, Novara, Italy

**Keywords:** titanium implants, multiparametric flow cytometry, immunobiocompatibility, acid etching, tissue regeneration, inflammation

## Abstract

**Introduction:**

While titanium and its alloys exhibit excellent biocompatibility and corrosion resistance, their polished surfaces can hinder fast and effective osseointegration and other biological processes, such as angiogenesis, due to their inert and hydrophobic properties. Despite being commonly used for orthopedic implants, research focuses on developing surface treatments to improve osseointegration, promoting cell adhesion and proliferation, as well as increasing protein adsorption capacity. This study explores a chemical treatment intended for titanium-based implants that enhances tissue integration without compromising the mechanical properties of the Ti6Al4V substrate. However, recognizing that inflammation contributes to nearly half of early implant failures, we assessed the impact of this treatment on T-cell viability, cytokine production, and phenotype.

**Methods:**

Ti6Al4V with extra low interstitial (ELI) content discs were treated with hydrofluoric acid followed by a controlled oxidation step in hydrogen peroxide that creates a complex surface topography with micro- and nano-texture and modifies the chemistry of the surface oxide layer. The acid etched surface contains an abundance of hydroxyl groups, crucial for promoting bone growth and apatite precipitation, while also enabling further functionalization with biomolecules.

**Results:**

While cell viability remained high in both groups, untreated discs triggered an increase in Th2 cells and a decrease of the Th17 subset. Furthermore, peripheral blood mononuclear cells exposed to untreated discs displayed a rise in various pro-inflammatory and anti-inflammatory cytokines compared to the control and treated groups. Conversely, the treated discs showed a similar profile to the control, both in terms of immune cell subset frequencies and cytokine secretion.

**Discussion:**

The dysregulation of the cytokine profile upon contact with untreated Ti6Al4V-ELI discs, namely upregulation of IL-2 could be responsible for the decrease in Th17 frequency, and thus might contribute to implant-associated bacterial infection. Interestingly, the chemical treatment restores the immune response to levels comparable to the control condition, suggesting the treatment’s potential to mitigate inflammation by enhancing biocompatibility.

## 1 Introduction

With the increased incidence and burden of musculoskeletal disorders, the development of improved biomaterials to be used as implants is of paramount importance (Cieza et al., 2020; Hossain et al., 2024; Abd-Elaziem et al., 2024). Although ceramics–such as alumina and zirconia–and polymers–e.g., ultra-high molecular weight polyethylene (UHMWPE) – generally possess some valuable characteristics as orthopedic implants, such as high wear resistance and low friction coefficients, these still fall short of metallic biomaterials in some applications, due to the importance of mechanical strength and toughness in ensuring safety (Szczęsny et al., 2022). Features such as high yield, fatigue and shear strength as well as plentiful hardness, ductility and fracture toughness along with biocompatibility advocate in favor of metal implants as an appropriate option for dental and orthopedic devices. On the other hand, there can be some drawbacks due to the reaction of body enzymes and acidic chemical environment during the inflammatory response on the implant surface that leads to the release of ions from the metallic surface. These ions can cause toxicity and bone resorption that may lead to implant failure, often due to aseptic loosening, and surrounding tissue damage (Katti, 2004; Davis et al., 2022; Fang et al., 2024).

The most common types of metallic biomaterials in use are Stainless-steel (SS), Titanium-based alloys, Cobalt-based alloys, Shape memory alloys (SMAs), and Magnesium-based alloys (Tanzi et al., 2019). In particular, titanium and its alloys are widely used in different biomedical applications such as orthopedic, cardiovascular, and dental implants without relevant toxicity issues (Ferraris et al., 2022). The choice for titanium generally implies low implant rejection (Petersen, 2014). The oxide layer on the surface of titanium implants confers resistance to corrosion and reduces ion release, contributing to its biocompatibility (Torrisi and Scolaro, 2017; Ferraris et al., 2011). The Ti6Al4V-ELI alloy was specifically developed for biomedical implants with improved corrosion resistance and fatigue strength due to the low amount of interstitial elements. However, due to being generally inert, the oxidized surface can lead to the formation of fibrotic tissue, which can impair osseointegration (Ferraris et al., 2011; Ratner, 2001).

Upon implantation, biomaterials swiftly become coated with proteins such as fibronectin, vitronectin, albumin, complement, among others, which adhere to the surface (Pitchai et al., 2023). This prompts activated platelets to release chemoattractants that guide the migration of macrophages to the wound site, where they adhere to the biomaterial surface through integrin-mediated interactions with the adsorbed proteins (Abaricia et al., 2021). Lymphocytes also appear at the implant site (Anderson, 2001) and they can also adhere to the surface and interact with macrophages. Consequently, the subsequent activation and modulation of lymphocyte function play a crucial role as an early element in the overall wound healing process following biomaterial implantation.

To complement the excellent mechanical properties of titanium with a more bioactive effect, surface modifications have been attempted, through techniques such as acid-etching, grit-blasting, particle sintering, plasma spray coating and micro-patterning (Ferraris et al., 2011; D’Esposito et al., 2021). In fact, a rough structure instead of a polished surface, greatly favors protein adsorption, formation of strong focal adhesions by osteoblasts, and implant stability (Le Guehennec et al., 2008). Many options are already available in the market, but their manufacturing methods may leave traces of harmful metal ions and other contaminants, which elicit immune reactions and impair osseointegration (Le Guehennec et al., 2008; Stricker et al., 2022). Moreover, there is an unmet medical need for multifunctional surfaces with fast osseointegration ability jointed to antibacterial action and immunomodulation ability (osseoimmunomodulation) (Spriano et al., 2018; Chen et al., 2016).

A recently optimized alternative to the currently used titanium-based implants has been developed at the lab scale through surface etching in diluted hydrofluoric acid, used to remove the native oxide layer, and then submerged in hydrogen peroxide to generate controlled oxidation (Ferraris et al., 2011). This creates a nanoporous titanium oxide layer with acidic hydroxyl groups, ideal for protein adsorption, which also possesses a high resistance to scratching (Ferraris et al., 2020a). This treatment has shown to be effective in the deposition of hydroxyapatite upon soaking in simulated body fluid (SBF), but also in reducing bacterial colonization, whilst improving osteoblast differentiation (Spriano et al., 2015; Ferraris et al., 2019). Surface features of the titanium implants in terms of exposed functional groups can also affect the immunomodulation process, especially in macrophages (Pitchai et al., 2022), but this topic is not largely investigated.

Considering that around half of early implant failures are due to inflammation (Gamna et al., 2023; Sun et al., 2017), it is essential to guarantee that any novel implant does not elicit an exacerbated inflammatory response. One of the currently open problems in orthopedics is the aseptic loosening of hip joint stems and cups originated by an inflammation process. It is a multifaceted issue influenced by several factors such as wear of the head of the prosthesis, implant design, material selection, and immune responses. The inflammatory response of the stem surface also plays a role. The here proposed etched titanium can be applied to hip stems and it is of interest to investigate its biological response in terms of inflammation. Therefore, we evaluated immune cell viability and cytokine production, as well as T-cell phenotype changes, induced by culture of peripheral blood mononuclear cells (PBMCs) in contact with titanium-based discs treated by acid etching and comparing with its respective untreated control.

## 2 Materials and methods

### 2.1 Titanium-based discs (Ti-6Al-4V–ELI) preparation

Titanium-based biomaterials, specifically the Ti-6Al-4V alloy with extra low interstitial (ELI) content (Grade 23, Titanium Consulting and Trading S.r.l., Firenze, Italy), were used. Ti6Al4V–ELI discs with 2 mm of thickness and 10 mm diameter were polished with SiC abrasive papers of 320, 600, and 800 grit attached to a sample holder and then polished manually with papers of 1,000, 2,500, and 4,000 grit. The samples were then washed with acetone in the ultrasonic bath for 10 min. Then, they were rinsed with ultra-pure water twice in the same condition and put to dry. Lastly, the sterilization was performed through autoclaving at 120°C for 20 min. These samples were subsequently referred to as “Polished”.

### 2.2 Surface chemical treatment (CT)

Attempting to improve osseointegration, we applied a patented surface treatment (EP2214732B1) to the titanium alloys, that consists in a thermochemical process that combines acid attack with surface oxidation (Spriano et al., 2007). Briefly, polished samples were soaked in 0.5 M hydrofluoric acid (HF, Sigma-Aldrich, St. Louis, MO, United States) to remove the native oxide layer, followed by a control oxidation step in 3.75 M hydrogen peroxide (H_2_O_2_, PanReac AppliChem, Darmstadt, Germany) with agitation. Lastly, the samples were rinsed with ultra-pure water, and then air dried and sterilized using UV for 1 h. This treatment confers a hydroxyl-enriched micro- and nanotextured oxide layer, intended to enhance apatite deposition and reduce bacterial adhesion (Ferraris et al., 2020a; Ferraris et al., 2019; Ferraris et al., 2020b; Alessandra Gobbo et al., 2023). These samples will be subsequently referred to as “Chemically Treated” or simply “CT”.

### 2.3 Sample physicochemical characterization

FESEM/EDS (FESEM - SUPRATM 40, Zeiss) was utilized to examine the surfaces of both polished and CT samples. Images were captured at magnifications of 60,000x and 1,50,000x. EDS analysis was conducted to quantify the atomic percentages (at-%) of titanium, aluminum, vanadium, and oxygen.

Surface wettability was assessed using static contact angle measurements via the sessile drop method (Krüss DSA 100, KRÜSS GmbH). Three measurements were performed on each sample, placing the liquid drops in different parts of the sample surface. Ultra-pure water served as the wetting fluid, with a 5 µL drop deposited on the surface using a pipette. Contact angles were measured using the instrument’s software (DSA-100, Dropshape Analysis, KRÜSS GmbH).

Zeta potential measurements were carried out with an electrokinetic analyzer (SurPASS 2, Anton Paar) equipped with an adjustable gap cell. The surface zeta potential was measured as a function of pH in a 0.001 M KCl electrolyte solution. Separate sets of specimen discs were used for the acidic and basic titrations to prevent artefacts from surface reactions during measurements. The acidic titration was performed by adding 0.05 M HCl, followed by basic titration with 0.05 M NaOH. Four parallel measurements were conducted for each pH point.

### 2.4 Blood specimen collection

Peripheral blood was obtained from seven healthy adult donors (4 females and 3 males, 25–45 years old, not under acetylsalicylic acid or nonsteroidal anti-inflammatory drugs administration) in cooperation with the Hospital Maggiore della Carità, Novara, Italy. From each donor, 10 mL of peripheral blood was withdrawn into lithium heparin collection tubes and immediately processed. The study was approved by the local ethics committee (prot. n. 675/CE).

### 2.5 PBMCs isolation

PBMCs were isolated from heparinized blood samples collected from healthy donors. The blood samples were mixed with equal amounts of phosphate buffer saline (PBS 1X) and were carefully overlaid on top of a density gradient isolation solution–Lympholyte-H (Cedarlane^®^, Ontario, Canada). After centrifugation at 1800 rpm, for 20 min, at room temperature, without acceleration/brake, the cell ring at the interface was collected, washed with PBS 1X, and cells were counted using a Neubauer chamber.

### 2.6 Apoptosis assay

To assess the effect of the Titanium-based discs on cell apoptosis, the eBioscience™ Annexin V-FITC Apoptosis Detection Kit (Invitrogen, Carlsbad, CA, United States) was used, substituting propidium iodide (PI) with 7-Aminoactinomycin D (Becton and Dickinson, NJ, United States) for its higher stability and specificity for DNA (Falzone et al., 2010; Olson et al., 2011). For this, PBMCs from three healthy adult donors were cultured in RPMI 1640, supplemented with 10% (v/v) heat-inactivated FBS, 100 U/mL penicillin/streptomycin, and 100 μg/mL gentamicin (Life technologies, CA, United States), at 37°C and 5% CO_2_. 1 × 10^6^ fresh cells/mL were seeded onto chemically treated or untreated Ti6Al4V–ELI discs for 48 h. Cells were harvested, washed with PBS 1X, and stained in accordance with manufacturer’s protocol. The stained samples were then acquired on FACSymphony™ A5 Cell Analyzer (Becton and Dickinson, NJ, United States) and analysed using BD FACSDiva software (Version 9.0., Becton and Dickinson, NJ, United States).

### 2.7 Immunobiocompatibility assay

PBMCs from seven healthy adult donors were cultured as previously described. 1 × 10^6^ fresh cells/mL were seeded onto chemically treated or untreated Ti6Al4V–ELI discs for 48 h. After media removal, cells were washed with PBS-EDTA 2 mM and stained with a viability dye–BD Horizon™ Fixable Viability Stain 780 – for 15 min, at 4°C. Cells were then washed with PBS-EDTA and Human BD™ Fc block solution was added. Antigen surface staining was performed by adding an antibody mix containing mouse anti-CD3 BUV496 monoclonal antibody (mAb) (clone: UCHT1; dilution 1:40), anti-CD4 BUV737 mAb (clone: SK3; dilution 1:400), anti-CD8 BUV805 mAb (clone: SK1; dilution 1:40), anti-CD25 APC-R700 mAb (clone: 2A3; dilution 1:40), anti-CD45 BUV395 mAb (clone: HI30; dilution 1:200), anti-CD127 BV786 mAb (clone: HIL-7R-M21; dilution 1:75), anti-CD45RA BUV563 mAb (clone: HI100; dilution 1:400), anti-CD183 APC mAb (clone: IC6; dilution 1:20), anti-CD194 PE-CF594 mAb (clone: 1G1; dilution 1:200), anti-CD196 BV480 mAb (clone: 11A9; dilution 1:40) and anti-CD197 BV711 mAb (clone: 150,503; dilution 1:40) in BD Horizon™ Brilliant Stain Buffer for 20 min, at 4°C. Lastly, the cells were washed and resuspended in PBS-EDTA for acquisition using a BD FACSymphony™ A5 flow cytometer. Data were then analyzed using the BD FACSDIVA™ software (Version 9.0., Becton and Dickinson, NJ, United States). All reagents were purchased from Becton and Dickinson (NJ, United States). The gating strategy is depicted as Supplementary Material (Supplementary Figure S1).

### 2.8 Enzyme-linked immunosorbent assay (ELISA)

PBMCs from five healthy adult donors were cultured as previously described. 1 × 10^6^ fresh cells/mL were seeded onto chemically treated or untreated Ti6Al4V–ELI discs for 48 h. Cell culture supernatants were collected after 48 h and cytokine levels were quantified using the Bio-Plex Pro Human Cytokine 17-plex Assay according to manufacturer’s instructions (Bio-Rad, CA, United States). This assay allows for the detection of a wide array of cytokines, specifically: G-CSF, GM-CSF, IFN-γ, IL-1β, IL-2, IL-4, IL-5, IL-6, IL-7, IL-8, IL-10, IL-12, IL-13, IL-17A, MCP-1, MIP-1β and TNF-α. The plate was run on a Bio-Plex 200 instrument (Bio-Rad, CA, United States). The reported concentrations and detection limits were obtained through the standard curves generated by the kit’s standards, using the weighted 5 PL curve fitting procedure in Bio-Plex Software Manager™. Values under the lower limit of quantification (LLOQ) were extrapolated based on the 5 PL logistic curve, as previously reported (Breen et al., 2016).

### 2.9 Statistical analysis

Data were analysed using repeated-measures (RM) one-way ANOVA or Friedman test with Bonferroni and Dunn’s post-hoc correction respectively, according to the sample’s normality, calculated using Shapiro-Wilk test. *p*-value below 0.05 was considered statistically significant. Statistical analyses were performed with GraphPad Instat software (Prism 8 version 8.4.3) (GraphPad Software, San Diego, CA, United States).

## 3 Results

### 3.1 Chemical and physical features of the surfaces

**FIGURE 1:**
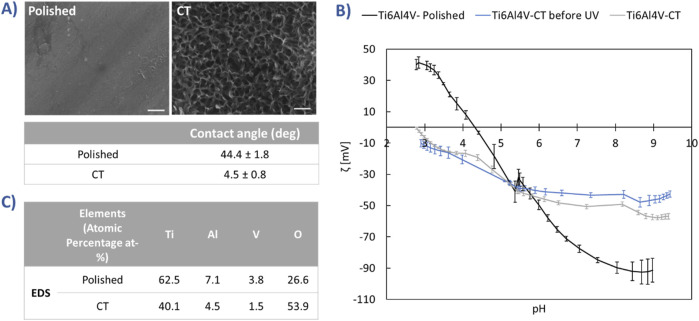
**(A)** The morphology (FESEM, scale bars 200 nm) and wettability of polished and CT surfaces; **(B)** Zeta potential titration curves of polished and CT, before and after UV treatment; **(C)** EDS elemental analysis of polished and CT samples.

CT surface was already described in detail from the chemical and physical standpoints and the main results are here summarized (Ferraris et al., 2020a; Ferraris et al., 2019; Gamna et al., 2023; Ferraris et al., 2020b; Alessandra Gobbo et al., 2023) in Figure 1. As shown in the FESEM images (Figure 1A), CT has a distinctive micro- and nanotextured surface structure, in contrast to the smooth, polished surface. Notably, CT exhibits a multiscale roughness along with high wettability (Figure 1A) and an abundance of acidic OH groups. This characteristic was further corroborated by zeta potential titration curves (Figure 1B). In the case of the polished sample, the isoelectric point (IEP) aligned with surfaces lacking functional groups with a strong acid-base reactivity. However, CT induced a shift in the IEP, indicative of the prevalence of OH groups with strong acidic behaviour. Moreover, the gentler slope of the curve underscored CT’s heightened wettability, as it suggests that water molecules adsorbed on the surface resisted displacement by ions in solution upon pH variation. No significant changes occurred before and after UV irradiation in terms of surface functional groups. Elemental analysis via EDS (Figure 1C) revealed the formation of a substantial titanium oxide layer, attributed to the chemical treatment.

### 3.2 Polished untreated and CT treated titanium-based discs do not affect viability of human PBMCs

**FIGURE 2:**
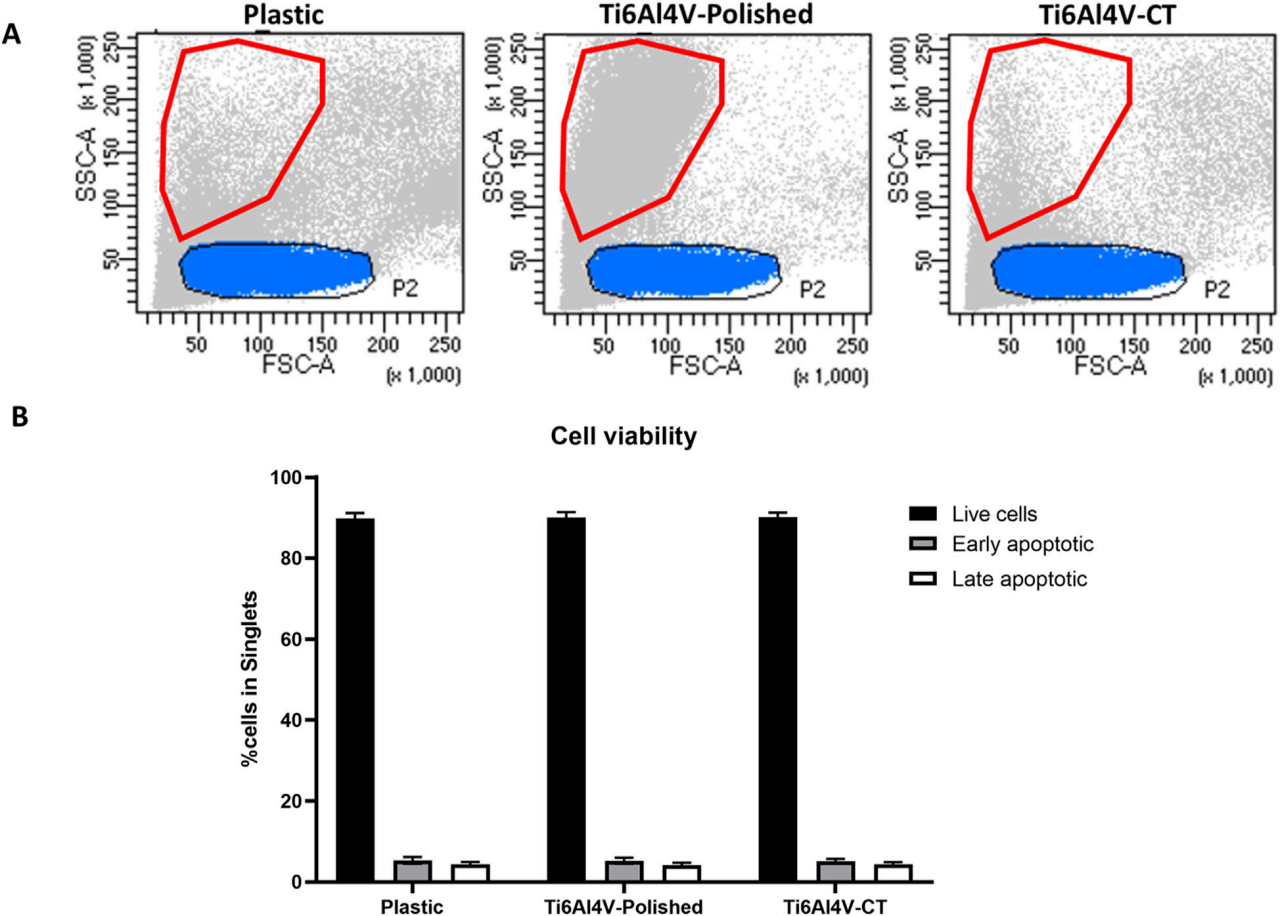
**(A)** Comparison between size (Forward scatter–FSC) and complexity (side scatter–SSC) of PBMCs cultured for 48 h in contact with either untreated (polished) or acid etched (CT), compared with the control (Plastic–no biomaterial); **(B)** Apoptosis assay of PBMCs cultured in contact with Ti6Al4V-ELI discs, through Annexin V/7-AAD assay. Bar graphs represent the percentages of live cells, identified as AnnV^−^/7-AAD^−^, cells in early apoptosis, as AnnV^+^/7-AAD^−^, and cells in late apoptosis, as AnnV^+^/7-AAD^+^.

PBMCs were cultured in contact with treated and untreated Ti6Al4V–ELI discs, and their cell viability was evaluated by flow cytometry. Firstly, we observed changes in the physical parameters when culturing PBMCs on titanium-based discs. Considering the size and complexity (Forward Scatter vs. Side Scatter, FSC vs. SSC) parameters, we found a population with smaller size (highlighted in red in Figure 2A) in Ti6Al4V–ELI polished samples, that does not exist in the control (plastic) or CT conditions. This effect was already observed at 24 h (data not shown) but it was exacerbated at 48 h. Lower light scattering as a result of cell shrinkage had been widely associated with apoptosis (Wlodkowic et al., 2012), thus we hypothesized that the polished condition could be leading to cell death. As shown in Figure 2B, we adapted a specific kit that allows for the distinction between live, early apoptotic, and late apoptotic cells. Specifically, Annexin V binds phosphatidylserine (PS), the main component of cell membranes which is typically not exposed in live cells, with complete integer cell membranes. Once apoptosis is induced, PS is translocated towards the external of the cell, thus being able to bind Annexin V, which due to being conjugated with FITC, becomes detectable through flow cytometry. Instead, the inclusion of 7-AAD, a non-permeable DNA intercalator, can only be present in late apoptotic cells. This is because only when the membrane loses its integrity later on the apoptotic phase, 7-AAD can penetrate the intracellular compartment and bind DNA in the nucleus (Zimmermann and Meyer, 2011). Altogether, the viability of the immune cells was not significantly affected upon culture with titanium-based discs up to 48 h, evidencing that although some cells become smaller, they are not undergoing apoptosis.

### 3.3 Multiparametric flow cytometry reveals a Th2/Th17 shift on polished untreated titanium-based discs

**FIGURE 3:**
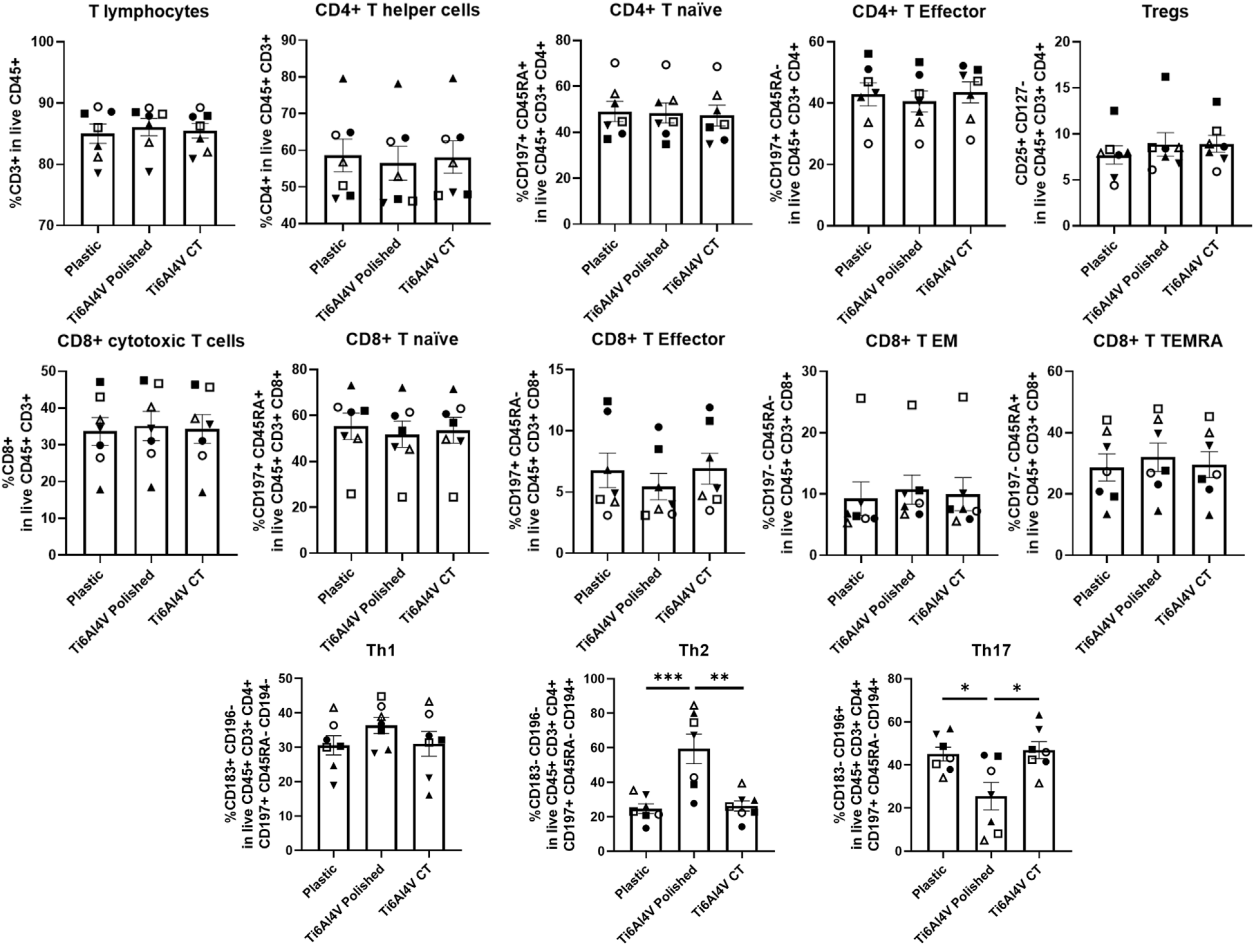
Immunophenotype of T cells cultured in contact with Ti6Al4V-ELI discs assessed through multiparametric flow cytometry. Scatter bar with plot graphs represent the percentages of immune cells after 48 h culture without biomaterial (cell culture plate plastic–control), with Ti6Al4V-ELI discs treated with acid etching (chemical treatment–CT) and its respective untreated discs (polished). Data are shown as average ±SEM, (n = 7). Each symbol represents a different donor. According to the data normality (Shapiro-Wilk test), RM one-way ANOVA (with Bonferroni post-hoc correction) or Friedman test (with Dunn’s post-hoc correction) were used. *p < 0.05, **p < 0.01., ***p < 0.001.

The immunophenotyping of PBMCs was performed upon their culture with polished or CT Ti6Al4V–ELI discs for 48 h, allowing for the distinction of several subsets of CD4^+^ T helper and CD8^+^ cytotoxic T cells. Although the tested biomaterials did not impact cell viability, we found a significant increase in the frequencies of Th2 cells, accompanied by the downregulation of Th17 cells, in the Ti6Al4V–ELI polished condition, when compared to the control (plastic) or the CT discs, as shown in Figure 3. Interestingly, we observed that the chemical treatment employed on the Ti6Al4V–ELI discs appears to revert the T-cell phenotype to a profile resembling the basal condition, where no titanium surface is present.

### 3.4 Polished untreated Ti6Al4V–ELI exacerbates PBMC cytokine-based responses

**FIGURE 4:**
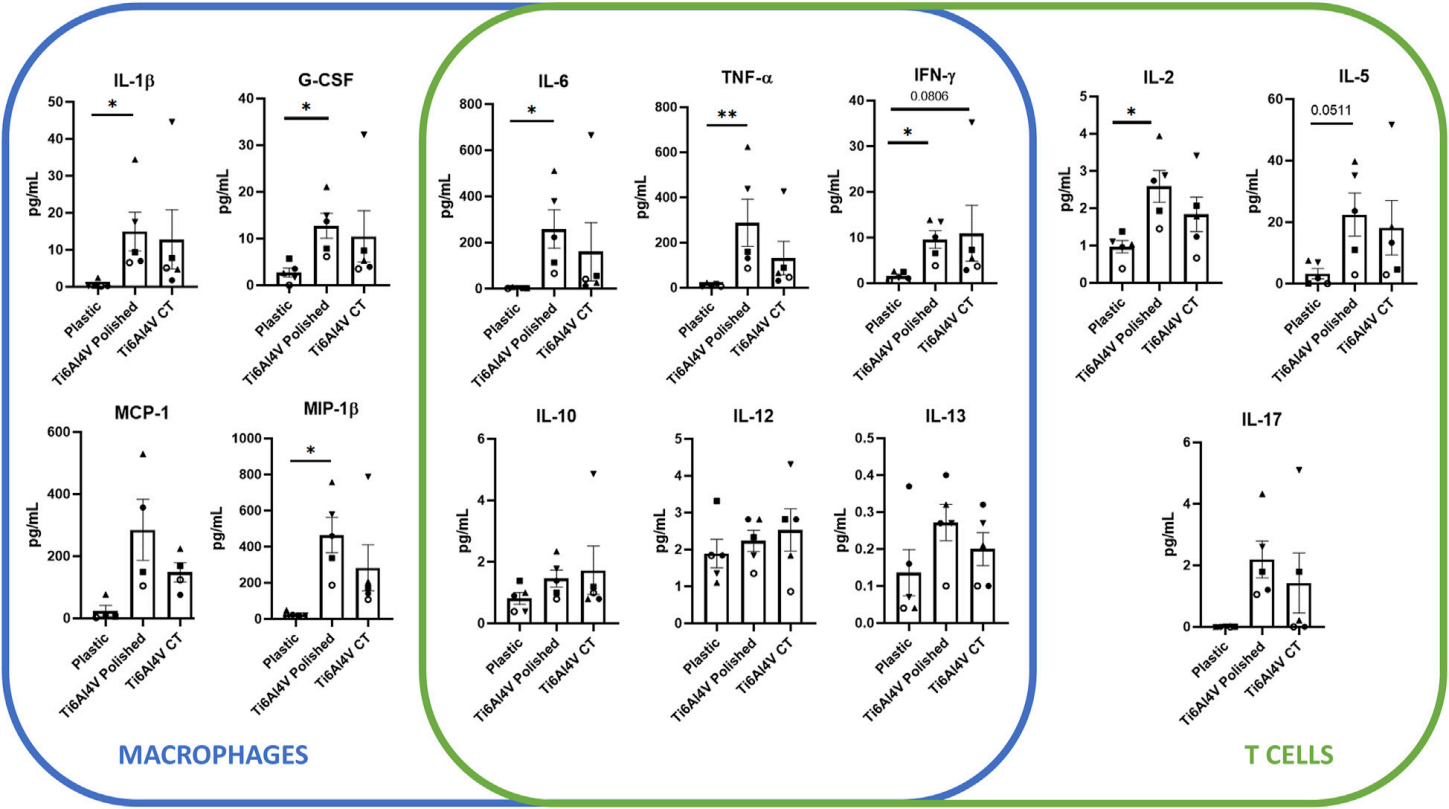
Cytokine expression levels of supernatants of PBMC after 48 h culture without biomaterial (cell culture plate plastic–control), in culture with Ti6Al4V-ELI discs treated with acid etching (chemical treatment–CT) and its respective untreated discs (polished). Data are shown as average ±SEM, (n = 5). According to the data normality (Shapiro-Wilk test), RM one-way ANOVA (with Bonferroni post-hoc correction) or Friedman test (with Dunn’s post-hoc correction) were used. *p < 0.05, **p < 0.01.

Due to these phenotypic differences found upon PBMC culture in contact with the different conditions, we questioned if polishing could also influence the secretion of both pro- and anti-inflammatory cytokines, evaluated by ELISA on the supernatants of PBMCs cultured in contact with the titanium-based discs for 48 h. Overall, among the conditions, the expression of GM-CSF, IL-4 and IL-7 cytokines were below the limit of detection of the kit. Although there were no detectable differences between CT and the polished discs, several cytokines were upregulated upon PBMC culture in contact with the polished discs in comparison with the basal control, more specifically G-CSF, IL-1β, IFN-γ, MIP-1β, IL-2, IL-6, and TNF-α. Particularly, in the polished condition the expression of IL-8 was over the upper limit of quantification, which may indicate a significant increase in the secretion of this cytokine, compared to the control and CT conditions (Figure 4).

## 4 Discussion

Several studies have investigated the biocompatibility of titanium and its alloys in the context of orthopaedic, cardiovascular, and dental implants (Verma, 2020). While targeting specific molecules with inhibitors, antibodies, or polysaccharides might be ideal for various diseases (Yu et al., 2022; Yi et al., 2023; Wang et al., 2024; Ge et al., 2023; Qiu et al., 2018; Zhang et al., 2023; Li et al., 2023; Niu et al., 2023; Zhang et al., 2024), patients with musculoskeletal disorders requiring replacement surgery could benefit more from the implantation of biomaterials with optimized surfaces. These biomaterials have the potential to modulate the immune response and enhance osseointegration in a broad and effective manner. Research has shown that titanium implants promote favourable biological responses, including minimal inflammatory reactions and the formation of a stable interface with the surrounding tissues (Stich et al., 2022).

In orthopaedic applications, titanium and its alloys are used to produce joint replacements, bone plates, and screws among the others. The mechanical properties of titanium, including its high strength and fatigue resistance, make it an ideal material for withstanding the load-bearing requirements of orthopaedic implants (Al-Shalawi et al., 2023). Furthermore, the biocompatibility of titanium allows for long-term implant stability and reduces the risk of adverse reactions within the body. Similarly, in dental implantology, these biomaterials are widely used for the fabrication of dental implants. The osseointegration capability of titanium allows for the direct structural and functional connection between the dental implant and the surrounding bone and soft tissue (gingiva), providing stability and support for dental prostheses, if the surface is properly treated (Nicholson, 2020). Additionally, the corrosion resistance of titanium ensures the longevity of dental implants within the oral environment (Kowalski et al., 2023).

Polishing plays a crucial role during the finalization and refinement of titanium-based products, since it removes surface irregularities and contaminants (Barbour et al., 2007), resulting in a smoother surface and corrosion resistance minimizing the risk of bacterial adhesion in niches due to roughness (Kowalski et al., 2023). Several studies have investigated the impact of polishing on human epithelial cells (Okubo et al., 2020), adipose stromal cells (Jung et al., 2020), and bone marrow-derived cells (Silva et al., 2009). Okubo et al. showed that the number of attached cells after 24 h of incubation exhibited a 60% decrease on polished surfaces, by compromising not only the attachment but also the retention of human epithelial cells (Okubo et al., 2020). Jung et al. reported that the polished-titanium discs may alter the expression of cellular matrix and focal adhesion genes (Jung et al., 2020). Silva et al. showed that adherent cells differentiate into osteogenic lineage on polished surfaces, though exhibiting a low proliferation activity (Silva et al., 2009).

In the present work, we aimed at investigating the influence of Ti6Al4V–ELI polished and CT discs on the initial cell responses and behaviour of human PBMCs, by evaluating any effects on cell viability, T-cell immunophenotype and cytokine release.

Our findings showed that both polished and CT Ti6Al4V–ELI discs did not affect cell viability of lymphocytes, while polishing altered the size of the cells that became smaller compared to the control and CT titanium discs. Our results align with those of Okubo et al. who reported that the size and perimeter of human epithelial cells were significantly reduced on titanium polished surfaces (Okubo et al., 2020), and they were restored upon ultraviolet light treatment. These authors concluded that this phenomenon is attributed to the adverse effects of the silicon carbide polishing of titanium surfaces. We hypothesize that the smaller cells observed on polished discs could correspond to monocytes, natural killer cells, and dendritic cells, which typically exhibit greater FSC than lymphocytes (gating P2, blue, Figure 2). These cells were possibly more sensitive to entering apoptosis upon contact with the Ti6Al4V–ELI discs without the chemical treatment. While polishing reduces surface roughness, it can also induce the release of nanoparticles (Song et al., 2021). Wang et al. reported that titanium particles *in vitro* may induce apoptosis of human mesenchymal stem cells (MSCs) (Wang et al., 2003).

While either polished and CT titanium discs did not affect cell viability or induced apoptosis of lymphocytes, we observed that polished titanium discs increased the percentage of Th2 cells while reducing Th17 cells, compared to both CT discs and plastic. However, both immune subsets were restored to levels like plastic when cultured with CT discs. In our recent work, we also observed a decrease in the percentage of Th17 cells in copper-doped bioactive glasses (Abreu et al., 2024), which was balanced by the increase of Th1 cells, together with the alteration in cytokines such as IL-5 and IL-13 and the chemokines MCP-1/CCL2 and MIP-1β/CCL4. We concluded that bioactive glasses may possibly modulate the immune response through cross-activation of cell types other than T lymphocytes, such as macrophages and eosinophils (Abreu et al., 2024). Th1 cells are associated with cell-mediated immunity and the production of pro-inflammatory cytokines, while Th2 cells are involved in humoral immunity and the secretion of anti-inflammatory cytokines (Berger, 2000). Th17 cells are characterized by the production of IL-17 cytokine and provide host protection against microbes that Th1 or Th2 immunity are not well suited for, such as extracellular bacteria and some fungi (Tesmer et al., 2008). The finding that polishing titanium discs may decrease the percentage of Th17 cells could, in part, explain the failure of titanium implants due to the development of bacterial infection (Souza et al., 2021). Among the cytokines that we have evaluated in the supernatant of cultured PBMCs, we did not observe any differences in IL-17 levels between polished and CT Ti6Al4V–ELI discs. However, there was a trend towards an increase of IL-17 levels in both polished and CT titanium discs compared to plastic, though the percentage of Th17 cell was reduced, this finding would suggest that other cell types may be responsible for IL-17 secretion. In fact, IL-17 can be also secreted by intraepithelial lymphocytes (IEL), present in the epithelial layer of the mucous membrane (Paroni et al., 2016), as well as γδT cells or NKT cells: the former, among PBMCs generally account for 1%–5% (Rimailho et al., 2023), while the latter between 5%–15% (Kokordelis et al., 2015).

We observed that IL-2 was upregulated in polished discs compared to plastic and it showed a slight increase compared to CT discs as well. IL-2 possesses dual and contrasting functions, i.e., it plays a role in both the initiation and resolution of inflammatory immune responses. IL-2 inhibits the development of Th17 cells (Hoyer et al., 2008) by also signalling via STAT5 (Laurence et al., 2007). We speculate that the decrease in Th17 cells observed in the polished sections was due to IL-2, which was then released in greater numbers.

Furthermore, we observed that polishing may also influence the release of other cytokines/chemokines. Regarding macrophages, G-CSF, IL-1β, and MIP-1β, were significantly upregulated in polished and with a trend in CT discs in comparison with plastic. G-CSF is a key regulator of neutrophils and it contributes to protecting the host against infection. Conversely, G-CSF can play a deleterious role in inflammatory diseases (Martin et al., 2021). IL-1β is a pro-inflammatory cytokine involved in several biologic processes, such as immune regulation, connective tissue metabolism, and inflammation among the others (Lopez-Castejon and Brough, 2011). As well as G-CSF, IL-1β also plays a protective role against the formation of bacterial biofilms, primarily by directly influencing the immune response to infections (Carta et al., 2006). MIP-1β/CCL4 is a chemokine which plays a crucial role in the recruitment of immune cells: our findings would suggest that both polished and CT titanium discs may promote the attachment of cells onto the surface, favouring the osseointegration (Gschwandtner et al., 2019).

On the T cells side, we found an increase of IL-5 levels in polished discs in comparison to plastic. These results align with the increase of Th2 cells, since IL-5 is produced by Th2 cells as well as innate immune ones with its main effects targeting eosinophil proliferation and promoting B-cell growth (Takatsu, 2011).

Lastly, we found that both polished and CT discs induced the secretion of IFN-γ and TNF-α cytokines. IFN-γ levels were increased in both polished and CT titanium discs, while TNF-α levels were increased only in polished discs in comparison with plastic. Both cytokines have a role in the fibrotic process, leading to the polarization of M1 macrophage (pro-inflammatory) and to the migration of fibroblasts to the biomaterial generating fibrosis (Yanez et al., 2017), which would ensure implant integration around the surrounding tissue.

In fact, the increased expression of TNF-α, IL-1β, IL-6 and IL-8, cytokines typically secreted by macrophages upon inflammation (Duque and Descoteaux, 2014), indicates a potential pro-inflammatory effect of the polished surface on this cell subset. This is further backed by the presence of a population of smaller cells with higher complexity than lymphocytes, which likely corresponds to macrophages, known to be contracted during the acute inflammation phase (Ronzier et al., 2022).

The dichotomy present on macrophage polarization is well-established and widely accepted, as M1 macrophages are associated with a pro-inflammatory profile, while M2 macrophages are deemed as anti-inflammatory. However, this does not render either subset as the sole contributor for tissue healing. A prolonged activity of M1 macrophages is known to impair the healing process (Sindrilaru et al., 2011); however, M1 macrophages and other cell types generally associated with supporting inflammation, such as Th1 cells, release cytokines (TNF-α, IFN-γ, IL-1β) that are crucial for immune cell and fibroblast recruitment and proliferation. On the other hand, M2 macrophages and Th2 are responsible for the resolution phase, counteracting the inflammation previously induced and facilitating fibroblast differentiation, angiogenesis and extracellular matrix deposition, mostly through the expression of several cytokines such as IL-4, IL-6 and IL-13 (Mantovani et al., 2004). Despite this, excessive activity of these cells leads to the formation of fibrotic tissue. As such, having only a pro- or anti-inflammatory response does not favor tissue regeneration or biomaterial integration. Overall, having a timely inflammatory reaction, followed by a balanced resolution phase is essential for tissue healing (Alvarez et al., 2016).

**FIGURE 5:**
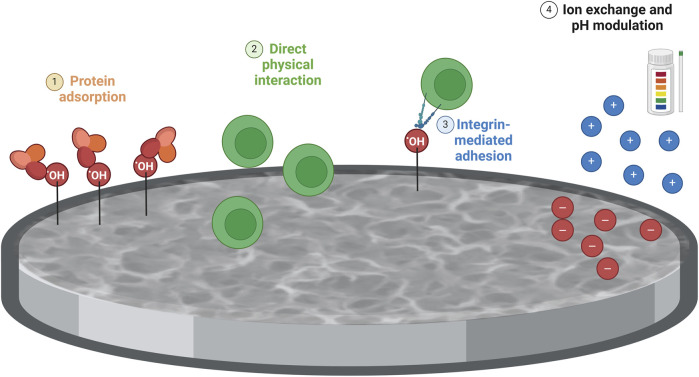
Schematic representation of the impact of the chemical treatment on immune cell response. The differences between polished and chemically treated Ti6Al4V-ELI samples might be due to surface alterations caused by the chemical treatment on Ti6AI4V-ELI, compared to polished discs, in terms of 1) Surface chemistry and reactivity: Chemical treatments can introduce reactive groups (e.g., hydroxyl groups) on the surface of the titanium alloy, enhancing protein adsorption. This improved adsorption facilitates integrin binding and activation of signaling pathways involved in T-cell activation and immune response; 2) Surface roughness and topography: Chemically treated surfaces typically exhibit increased roughness and more complex topographies compared to polished surfaces. This increased roughness, by providing more surface area and physical cues for cells, could represent a stimulus for T cells; 3) Increased surface roughness and reactive sites on chemically treated surfaces improve integrin-mediated adhesion and activate downstream signaling pathways (e.g., MAPK, PI3K/Akt) that could regulate cytokine production; 4) The surface characteristics of chemically treated samples create a favorable microenvironment for cells by modulating ion exchange, pH, and biochemical milieu, which could influence cytokine production. Created with Biorender.com.

Chemical treatments often increase surface roughness and create micro- or nanoscale topographical features (Gittens et al., 2011). These increased surface features can enhance cell attachment, spreading, and proliferation, thereby stimulating cytokine production due to more significant cellular interactions and signaling. Chemical treatments can also modify the surface chemistry of Ti6Al4V–ELI samples by introducing new functional groups or changing the oxidation state of the surface (Alessandra Gobbo et al., 2023; Karlsson et al., 2015; Lallukka et al., 2022). These chemical changes can enhance protein adsorption and cellular interactions. Improved protein adsorption facilitates better integrin binding and activation of signaling pathways that lead to the cytokine production. The physical characteristics of chemically treated surfaces can induce different mechanical stresses on adhering cells (Metwally and Stachewicz, 2019). Cells respond to these mechanical cues by altering their cytoskeletal organization and signaling pathways, which can lead to variations in cytokine production. The surface characteristics of chemically treated samples can create a more favorable microenvironment for cells, including changes in ion exchange, pH, and local biochemical milieu (Jiang et al., 2023). These factors can modulate cellular behavior and cytokine production (Figure 5).

A recent study has demonstrated that roughness and wettability of titanium implants influences the recruitment of several cell types in mice, such as neutrophils, macrophages and MSCs. Moreover, the knockdown of either CD4^+^ or CD8^+^ T cells can differentially modulate cell recruitment, polarization and proliferation, which showed diverse effects depending on the implant’s surface characteristics (Avery et al., 2024). Besides the widely studied impact on macrophages, our results confirm that T cells can suffer substantial phenotypical changes due to surface modifications on titanium implants. Given that T cells are do not adhere to substrates, roughness and wettability of the discs should not affect T-cell behaviour. On the other hand, the cytokine profile exhibited by PBMCs upon culture with polished or CT discs highlights how the surface composition, particularly the presence of OH groups might alter its cytokine production, therefore also its inflammatory profile.

While direct culture of fibroblasts, osteoblasts and macrophages might give important insight on how the organism reacts to an implant, our study demonstrates that T cells, although they are not expected attach to the modified surfaces, suffer phenotypical changes upon culture with titanium-based discs. Given the crucial role of T cells on inflammatory modulation, fibrosis, and cell recruitment (such as mesenchymal stem cells), it becomes increasingly relevant to study the impact of optimized biomaterials for musculoskeletal regeneration on these cells (Avery et al., 2024; Sun et al., 2023), although data regarding the impact of Titanium-based implants on PBMCs is still scarce (Supplementary Table S1). Even though polished surfaces may exhibit good biocompatibility, chemically treated surfaces can enhance the biological response by promoting beneficial cell behaviours and reducing adverse reactions (Ferraris et al., 2011). This improved biocompatibility can reduce the risk of implant rejection and inflammation (Rahnama-Hezavah et al., 2023). Moreover, the presence of cytokines in the microenvironment surrounding the implantation site, potentially produced by the aforementioned cell types, can lead to a differential activation of T helper subset; in turn, each specific subset produces a set of effector cytokines, which can aggravate or ameliorate the inflammatory and fibrotic processes by cross-activating several cells types, including macrophages and neutrophils, which can bring severe health issues to the patient (Owen et al., 2019). Overall, while studying the impact of biomaterials on the cells that are directly in contact with the implant is undoubtedly relevant, adaptive immunity cells such as T cells should not be overlooked, as they are capable of influencing the host’s response to the implant and contribute to proper osseointegration. Thus, the primary objective of our study was to explore the immunomodulatory effects of titanium-based biomaterials on PBMCs, more specifically on T cells. By focusing on the interaction between solely PBMCs and the biomaterials, we aimed to establish a foundational understanding of their immune responses before progressing to more complex models involving bone remodeling. Considering the important role played by mesenchymal stem/stromal cells (MSCs) and osteoblasts in reshaping the bone microenvironment, future works should focus on assessing the behavior of these cell types when co-cultured together with PBMCs in contact with polished or etched Ti6Al4V–ELI surfaces.

In conclusion, the proposed acid etching treatment did not harm immune cells’ viability, while being able to mitigate the increase in Th2 cells and a decrease of the Th17 subset resulting from the polished surfaces. Moreover, polishing creates an imbalance in the pro-inflammatory and anti-inflammatory cytokine profile produced and secreted by PBMCs, compared to the control and treated groups; this condition also leads to the presence of a population with smaller size but higher complexity than lymphocytes, which altogether could indicate the activation of macrophages in the acute phase of inflammation. Conversely, the chemically treated discs showed similar results to the control condition without biomaterial, suggesting the treatment’s potential to mitigate inflammation by enhancing biocompatibility.

Overall, it is important to consider that the polishing process may not only alter the surface topography of biomaterial, but also impact the initial response of immune cells, which refers to the immediate reaction of the body’s immune system upon contact with it. This includes processes such as inflammation, immune cell recruitment, and cytokine release among the others, which are pivotal in determining the biocompatibility and suitability of the biomaterial for its intended application. Understanding this initial response is essential for assessing the potential immunogenicity and overall compatibility of the biomaterial within the biological environment. CT of titanium discs may offer a solution to mitigate the effects observed with polished titanium discs, thus holding significant relevance in the context of biomedical applications.

## Data Availability

The original contributions presented in the study are included in the article/Supplementary Material, further inquiries can be directed to the corresponding author.
