# Supplementary material for: Evaluation of the immune response of peripheral blood mononuclear cells cultured on Ti6Al4V-ELI polished or etched surfaces
Source: Front Bioeng Biotechnol. 2024 Oct 8;12:1458091. doi: 10.3389/fbioe.2024.1458091 (PMC11493608; doi:10.3389/fbioe.2024.1458091)
Supplement: Supplementary file 1 [file DataSheet1.pdf]

Supplementary Material

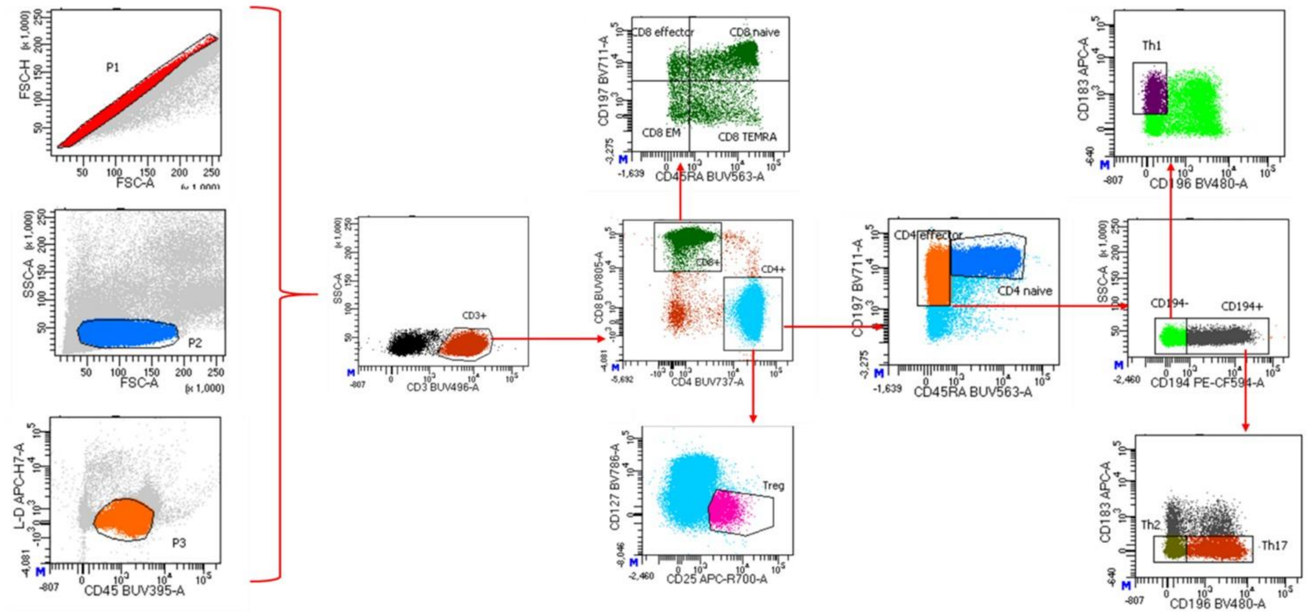

Supplementary Figure 1. Gating strategy. PBMCs were stained with the following antibodies: monoclonal anti-CD45 human antibody (mAb) (leucocytes), anti-CD3 mAb (T cells), anti-CD4 mAb (T helper cells), anti-CD8 mAb (cytotoxic T cells) anti-CD45RA and anti-CD197 mAbs (naïve, central memory (CM), effector memory cells (EM), and terminally differentiated EM cells), anti-CD25 and anti-CD127 mAbs (regulatory T cells, Tregs), anti-CD183, anti-CD194 and anti-CD196 mAbs (Th1, Th2, and Th17). The samples were acquired BD FACSymphony™ A5 flow cytometer. Data were analyzed using the BD FACSDIVA™ software (Version 9.0., Becton and Dickinson, NJ, USA).

Supplementary Table 1. Comparison of the methodology and main findings with similar studies.

Culture duration	PBMC density	Material	Findings	Reference
**24 and 48h**	2 x 10^6 PBMCs/sample	Ti6Al4V	↑ TNF-α in polished discs compared with control without discs	(1)
**72h**	7.5 x 10^5 PBMC/sample	Straumann Titanium-based dental implants	↑ IL-6 and IL-8 in acid etched discs compared with control without discs	(2)
**24, 72h and 6 days**	2 x 10^6 PBMCs/sample	Commercially pure titanium	↑ GRO-α, MIG, MCP-1, IL-13, IL-4, -6, -8, -1β and TNF-α in polished titanium compared with treated discs (titanium oxide nanotubes on the surface)	(3)
**24h**	1 x 10^6 PBMCs/mL	Ti6Al4V	↑ IL-8 in polished discs	(4)
**5 days**	1 x 10^6 PBMCs/mL	Etched Titanium (commercially available)	↑ IL-6, GRO-α (CXCL1), ENA-78 (CXCL5) and EGF in etched discs compared with control without discs	(5)
**24h**	5 x 10^4 PBMCs/sample	Alumina-blasted and acid-etched (AB/AE) Ti6Al4V alloy	↓ IL-1B in AB/AE alloy compared with control without discs	(6)
**6 days**	2 x 10^6 PBMCs/mL	Polished medical-grade Titanium discs	↑ MMP9 in whole PBMCs (but not in PBMC without CD14+ monocytes) in polished discs compared with control without discs	(7)

1. Martinesi M, Bruni S, Stio M, Treves C, Borgioli F. In vitro interaction between surface-treated Ti-6Al-4V titanium alloy and human peripheral blood mononuclear cells. J Biomed Mater Res - Part A. 2005;74(2).

2. Billing F, Jakobi M, Martin D, Gerlach K, Arefaine E, Weiss M, et al. The immune response to the SLActive titanium dental implant surface in vitro is predominantly driven by innate immune cells. J Immunol Regen Med. 2021;13.

3. Östberg AK, Dahlgren U, Sul YT, Johansson CB. Inflammatory cytokine release is affected by surface morphology and chemistry of titanium implants. J Mater Sci Mater Med. 2015;26(4).

4. Schildhauer TA, Peter E, Muhr G, Koller M. Activation of human leukocytes on tantalum trabecular metal in comparison to commonly used orthopedic metal implant materials. J Biomed Mater Res - Part A. 2009;88(2).

5. Himmlova L, Kubies D, Hulejova H, Bartova J, Riedel T, Stikarova J, et al. Effect of Blood Component Coatings of Enosseal Implants on Proliferation and Synthetic Activity of Human Osteoblasts and Cytokine Production of Peripheral Blood Mononuclear Cells. Mediators Inflamm. 2016;2016.

6. Moura CG, Souza MA, Kohal RJ, Dechichi P, Zanetta-Barbosa D, Jimbo R, et al. Evaluation of osteogenic cell culture and osteogenic/peripheral blood mononuclear human cell co-culture on modified titanium surfaces. Biomed Mater. 2013;8(3).

7. Gudima A, Hesselbarth D, Li G, Riabov V, Michel J, Liu Q, et al. Titanium induces proinflammatory and tissue-destructive responses in primary human macrophages. J Leukoc Biol [Internet]. 2024 Mar 21;qiae072. Available from: https://doi.org/10.1093/jleuko/qiae072
